# Glyphosate residues in soil affect crop plant germination and growth

**DOI:** 10.1038/s41598-019-56195-3

**Published:** 2019-12-23

**Authors:** Marjo Helander, Anna Pauna, Kari Saikkonen, Irma Saloniemi

**Affiliations:** 1 Department of Biology, University of Turku, 20014 Turku, Finland; 2 Biodiversity Unit, University of Turku, 20014 Turku, Finland

**Keywords:** Ecology, Environmental sciences

## Abstract

Glyphosate-based herbicides (GBH) are the most widely used pesticides globally. Their persistence in soils and effects on non-target organisms have become a concern in agricultural and natural ecosystems. We experimentally studied, whether residues of GBH (Roundup Gold) or pure glyphosate in soils affect the germination or sprouting and growth of crop plants after the safety period. The seed germination of faba bean, oat and turnip rape, and sprouting of potato tubers was delayed in the greenhouse experiments in soils treated with GBH or with pure glyphosate. The total shoot biomass of faba bean was 28%, oat 29% and turnip rape 58% higher in control compared to GBH soils four weeks after sowing. In the beginning of the growing season, the plant growth in the field experiment supported the observations in the greenhouse experiment. However, at the end of the field experiment, potato shoot biomass was 25% and tuber biomass 14% greater in GBH soil compared to control soil. Potato tubers tended to gather low amounts of glyphosate (0.02 mg/kg) and its metabolite AMPA (0.07 mg/kg). Grazing by barnacle geese was three times higher in oats growing in the GBH soils compared to control oats in the field. Our results draw attention to complex indirect effects of GBH on crop plant seedling establishment and resistance to herbivores.

## Introduction

In agricultural fields, the principal barriers to seedling emergence and establishment are physical hazards (e.g., desiccation, seed predation and herbivory)^1^. Unlike in natural environments, in crop fields, farmers control the abiotic and biotic conditions to provide an optimal environment with minimal economical costs and work contribution. Nutrient availability in agronomic fields is usually optimized for the selected crop species or cultivars, and competition among crop plant individuals is prevented by sowing the seeds in the preferred quantity. Furthermore, competition between crop plants and weeds is managed either by tilling the soil or by herbicide treatments.

Glyphosate-based herbicides (GBH) became the most popular herbicides worldwide after the patent expired in 2000^2,3^. Today, GBHs are the cheapest and most efficient herbicides, and they have played a central role in the development of agricultural practices during recent decades. Most fields are treated with glyphosate either before sowing in the spring or after harvesting in the fall. Moreover, minimum and no-tillage cropping, where seeds are sown under glyphosate-treated vegetation, has increased glyphosate use^4^. In addition, the common desiccation of cereal, bean and seed crops with GBH prior to harvest as well as GBH applications in silviculture and horticulture are adding to the consumption of what is now the most widely used weed killer in the world^2^. In countries where genetically modified crop plants like corn, soybean and canola are grown, glyphosate resistance is the major method for enabling the cultivated fields to be sprayed with herbicides for weed control during the growing season^5^.

The efficiency of glyphosate is based on its ability to inhibit the enzyme 5-enolpyruvylshikimate-3-phosphate (EPSP) synthase, thus blocking the synthesis of three essential amino acids, phenylalanine, tyrosine and tryptophan, of the shikimate pathway. This, in turn, prevents the plant from producing compounds that play crucial roles in the plant’s growth, development, reproduction, defense and environmental responses^6,7^. All plants and most microbes^8,9^ have the shikimate pathway, but it is never present in animal cells^7^. Hence, it is presumably non-toxic to humans and animals^10^. However, this assumption has been questioned recently^11,12^.

Glyphosate is assumed to degrade rapidly in the soil through microbial transformation to aminomethylphosphonic acid (AMPA) or sarcosine^13^. Comparable to glyphosate treatments, AMPA has been shown to decrease photosynthetic activity and increase peroxide accumulation due to the inhibition of antioxidant enzyme activities^14^. In contrast to the general supposition that GBH degrades rapidly, observations especially in northern ecosystems have demonstrated that glyphosate degradation in cold environments may be delayed, and glyphosate remnants may be detected months or years after the last application^6,15–19^.

In this study, we experimentally investigate whether GBH remains in soils affect crop plant establishment and growth in greenhouse and field conditions in the boreal zone. To separate the possible adjuvant effects of the commercial GBH products from those of pure glyphosate, we performed a greenhouse study in which these two treatments were applied separately to the soil two weeks before crop sowing or planting.

## Methods

### Plant material

As model plants we used four commonly cultivated crop plants in Finland: faba bean (*Vicia faba* L.) cultivar ‘Kontu’, oat (*Avena sativa* L.) cultivar ‘Iiris’, turnip rape (*Brassica rapa* subsp. *oleifera* (DC.) Metzg.) cultivar ‘Apollo’ and potato (*Solanum tuberosum* L.) cultivar ‘Ditta’. The faba beans and potatoes were organic, while the turnip rape and oat seeds were from conventional farms.

### Greenhouse experiment

To specifically study the germination success and early seedling establishment, separating the glyphosate effects from the adjuvant effects in the commercial GBH, we conducted a greenhouse experiment in the University of Turku Ruissalo Botanical Garden research greenhouse in July–September 2016. Faba beans, oats, turnip rape and potatoes were seeded or planted in control soil (C), commercial herbicide (Roundup Gold)-treated soil (GBH) or glyphosate-treated soil (G).

The soil used in the greenhouse experiment was organic seedling soil (Biolan kylvö- ja taimimulta, www.biolan.fi; compost soil with sand and turf, pH 6.2, N 100 mg/l, P 70 mg/l, K 400 mg/l with added mycorrhiza inocula *Glomus intraradices* 40 spores/l and *Gliocladium* sp 108 spores/l). For control treatment (C), 48 liters of the soil was grinded in a cement mixer and 4 liters of tap water was carefully added to the soil. The G and commercial GBH treatments were proceeded in the same way as the control treatment, except that for the G treatment we added 250 mg of pure glyphosate (Pestanal, (HO)_2_P(O)CH_2_NHCH_2_CO_2_H, molecular weight 169,07 g/mol) to tap water, and for GBH treatment we added a corresponding amount of Roundup Gold to equal 250 mg of glyphosate, to approximately correspond the amount of glyphosate added to the field experiment. According to the manufacturer’s instructions, glyphosate in the soil degrades in 10–14 days. Thus treated soils were allowed to stand in the greenhouse conditions for 14 days. Then, the pots (5 cm × 5 cm × 5 cm for faba beans, oats and turnip oil and 13 cm × 13 cm × 13 cm for potatoes) were filled with treated soils, and two faba beans, four oat seeds, four turnip rape seeds and one potato tuber (mean weight 30 g) were planted in marked pots. All the crop species and treatments were repeated 15 times, and the pots were randomized to five tables (blocks) in the greenhouse.

The minimum temperature in the greenhouse was set to 18 °C, but during the daytime the temperature rose to 24 °C. Air humidity was set to 70%, and day length was the ambient August day length in southwest Finland (about 15 h). The pots were watered as needed during the experiment.

The germinating plants in the pots were recorded, and their height was measured seven times during the next three weeks. The experiment was terminated four weeks from sowing by clipping the shoots of the plants 2 cm above ground, and drying (2 days, 65 °C) and weighing the biomass. Turnip rape plants from one pot were combined and weighed together, but faba beans, oats and potatoes were treated as individual plants.

Samples for glyphosate analyses were taken from each soil treatment (control, pure glyphosate, GBH; 200 g of bulk soil/treatment) two days after the treatments. To analyze possible glyphosate traces in plants, 5–10 shoots/treatment/crop species were collected in the end of the experiment. The air-dried plant and soil samples were sent for glyphosate and AMPA analysis to GroenAgro (www.agrocontrol.nl/en/). Extraction was performed with a mixture of water and acidified methanol. The analyses were performed with a liquid chromatography coupled to a tandem quadruple mass spectrometer (LC-MS/MS). The separation was performed with a mix mode column using a gradient based on mixture of water and acetonitrile. Two specific MRM’s (multiple reaction monitoring) were used to identify the component and standard addition to quantify the concentration.

### Field experiment

The field experiment was conducted in the University of Turku Ruissalo Botanical Garden in southwest Finland (60°26′N, 22°10′E). The mean annual temperature (30-year mean) in the area is 5.7 °C, and the mean annual precipitation is 690 mm (http://www.fmi.fi/en). Months-long frost temperatures, snow cover and short days (<6 h in January) characterize the winters in the area. In summer, the long-term mean temperature is 16 °C, the precipitation is 211 mm and longest day is 19 h (in June).

The experiment was established in 2013. Sand and peat were spread in the experimental area (25 m × 50 m) to enhance the soil quality before tilling to a depth of 15 cm. The soil type in the field was medium clay with high organic matter content (after peat addition >120 g kg^−1^) and a pH of 7.1. The field site had no previous history of herbicide applications.

In spring 2014, the experimental field was divided into alternating 10 C and 10 GBH treatment plots (23 m × 1.5 m), with 1.5 m buffer strips between the plots. The buffer strips were mowed several times during the field seasons to minimize weed invasion. Twice a year (May 2014, 2015 and 2016; October 2014 and 2015) the plots were first tilled to a depth of 5 cm using a hand rotary tiller. Then, the C plots were treated with tap water (5 l/plot) and the GBH plots with Roundup Gold (glyphosate concentration 450 g l^−1^, CAS: 3864-194-0, application rate 6.4 l ha^−1^ in 5 l of tap water per plot). We used the maximal, but still realistic permitted glyphosate dosage (3 kg ha^−1^). The treatments were applied with a hand-operated pressure tank, using a plastic hood in the sprinkler tip to prevent the glyphosate from spreading outside the treatment plots. During the growing season, both the C and GBH plots were hand weeded to keep the soil structure and plant competition as similar as possible in all plots.

**Figure 1:**
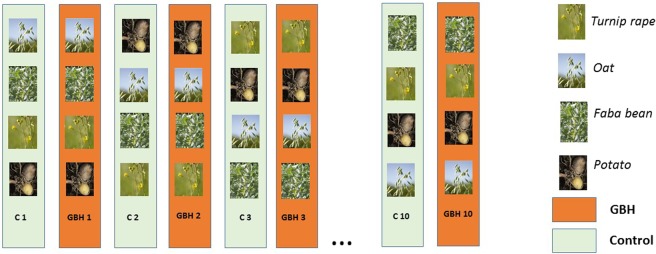
In the field experiment oats, turnip rapes and faba beans were sowed and potatoes planted to sub-plots in ten control (C) and ten glyphosate based herbicide (GBH) plots.

In June 2016, three weeks after the GBH and C treatments, the plots were prepared for sowing and planting. Four sub-plots (1.5 m × 1.5 m, 0.5 m buffer strip between the sub-plots) from each plot were randomized to four crop species (faba bean, turnip rape, oats, potato) so that adjacent GBH and C plots had identically assigned sub-plots (Fig. 1). Common seedling rates were used: about 80 faba beans (40 g), 600 oat grains (27 g) and 300 turnip rape seeds (0.70 g) were sown, and 16 potato tubers (mean 30 g) were planted in 4–6 rows in each plot. The plots were watered twice during the growing season and hand weeded to remove the competing vegetation.

The plants were measured four times, first time 6 weeks and last time 10 weeks after the sowing and planting. From each faba bean and oat sub-plot, 20 plants were randomly marked as experimental plants to be measured individually during the study. Because of the poor germination of the turnip rape seeds in the field, all the emerging plants were measured. Furthermore, all 16 potato plants in each plot were measured during the study. The height of the faba beans, oats, turnip rapes and potatoes was measured with a measuring tape. Eleven weeks from sowing and planting, all the experimental faba beans, oats, potatoes and turnip rapes were cut 2 cm above-ground and transported to the lab. The above-ground parts of all plants were oven dried (two days, 65 C°) and weighed. All potato tubers were collected, gently cleaned with a brush and weighed.

Six weeks after sowing, the oat sub-plots were grazed by barnacle geese. The birds were observed in the plots, and their droppings were detected in the oat sub-plots. Herbivore damage in the oat sub-plots was estimated by allocating the experimental plants to three classes (0 = no damage, 1 = moderate damage, 2 = high damage), and the number of flowering oats was counted.

At the end of the experiment, the lowest leaf was collected from each experimental plant for glyphosate analyses. In addition, potato tubers (25–50 g) were collected for further glyphosate analyses.

Soil samples (5 soil cores, 2.5 cm in diameter and 5 cm in depth) from each experimental plot were taken for glyphosate analyses in May and July 2016. The plant and soil material was air-dried before it was sent for analysis. Due to the high cost of glyphosate analysis, samples collected from different plots were pooled before the analysis. Soil and plant glyphosate and AMPA concentrations were analyzed by GroenAgro (www.agrocontrol.nl/en/). Analyses were performed the same way as the samples from the greenhouse experiment.

### Statistical analyses

The number of seeds germinated out of the total number of seeds planted per pot (faba bean, 2 seeds; oat and turnip rape, 4 seeds) per day in the greenhouse experiment was analyzed using generalized mixed linear models, with binomial distribution and logit link and block (table in greenhouse) as a random factor (function glmer in R package lme4). The cumulative number of germinated seeds out of all seeds was analyzed based on time (day), soil treatments (C, G, GBH) and their interaction (interaction was omitted from the final models, as it was not statistically significant). After the analysis, the three treatments (C, G, GBH) were compared using Tukey tests (function glht in R package multicomp). The total number of germinated seeds by the end of the experiment (last day recorded) was analyzed separately using the same procedure with the predictor ‘day’ omitted.

The number of potato sprouts in the greenhouse was analyzed the same way as the seed germination, except that the Poisson distribution with log link was used. The final number of potato sprouts in the greenhouse and the number of faba bean pods and oat flowers in the field experiment were also analyzed in the same way (without ‘day’).

The biomass and height of the plants both in the greenhouse and in the field were analyzed using linear mixed models (function lme in R package nlme), taking the random block (plot in the field and table in the greenhouse) effect into account and using the treatment (C, GBH, in greenhouse experiments also G) effect as a predictor. Differences between treatments were compared using Tukey tests in R program emmeans. The biomass of faba beans was log transformed, and the length of the oats was square-root transformed to gain normality.

The oats in the field experienced herbivore damage that killed or slowed down plant growth and resulted in serious deviances from normality. Chi-square tests were first used to compare the number of oats that survived and were damaged by herbivores. Survival of the plants until end of the experiment was analyzed using generalized mixed linear models (glmer), with treatment and herbivore damage level as predictors, plot as random and assuming a binomial distribution. The growth of the non-damaged oat plants was analyzed similarly to biomass and height above. When analyzing oat growth, herbivore damage level was included as a predictor.

Significance levels were calculated using the ANOVA function (package car).

All the figures are drawn using raw data from the experiments, while the statistically significant differences between the treatments in the figures are from the statistical models.

## Results

### Faba beans

**Figure 2:**
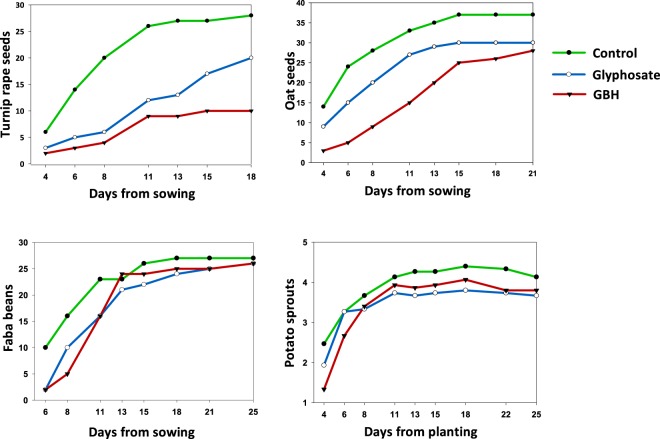
Germinated turnip rape (60 seeds/treatment), oat (60 seeds/treatment) and faba beans (30 beans/treatment) and sprouted potatoes (15 potatoes/treatment) in soils treated with glyphosate-based herbicide (GBH, Roundup Gold), glyphosate or control soils in the greenhouse experiment. Figures are drawn using raw data from the experiments.

**Figure 3:**
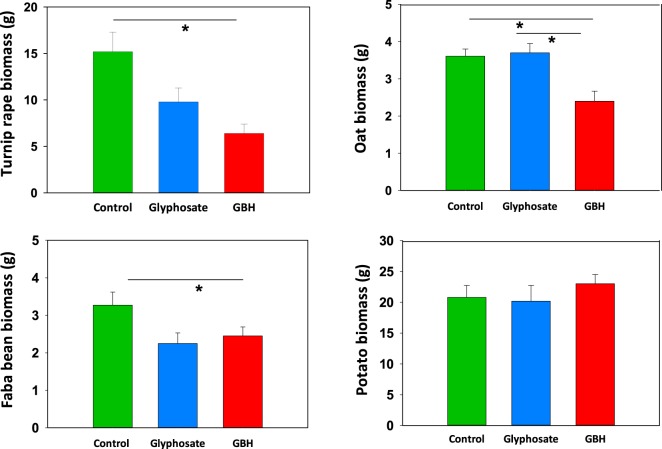
Above-ground biomass (mean ± S.E.) of turnip rapes, oat, faba bean and potato growing in soils treated with glyphosate-based herbicide (GBH, Roundup Gold), pure glyphosate or control soil. The seeds were sown after two weeks safety period from the soil glyphosate treatments. Biomass was collected four weeks after sowing the seeds. Figures are drawn using raw data from the experiment. Turnip rape plants from one pot were combined and weighed together, but faba beans, oats and potatoes were treated as individual plants. Statistically significant differences between treatments (*p*-values < 0.05) are marked with *.

In the greenhouse experiment, faba beans germinated faster in C soil than in soil treated with GBH (Fig. 2), but the germination rate did not differ between pure G and GBH soils (Table 1). Total germination success (83–90%) did not differ between the treatment groups after 25 days (χ^2^ = 0.29, df = 2, *p* = 0.754). At the end of the experiment, the above-ground biomass of the beans differed between the treatments (Fig. 3; χ^2^ = 7.53, df = 2, *p* = 0.023). The C plants were heavier compared to the GBH plants (post hoc: C–GBH, t = 2.57, *p* = 0.041; C–G, t = 2.10, *p* = 0.109; G–GBH t = 0.51, *p* = 0.868, df = 27).

**Table 1:** Germination (faba bean, oat, turnip rape) and sprouting (potato) rate in the greenhouse experiment.

	Treatment	Tukey comparison	C–G	C–GBH
	C, G, GBH	G–GBH		
Faba bean	χ2 = 18.33, df = 2, ***p*** < **0**.**001**	z = 0.24, *p* < 0.968	z = 3.66, ***p*** < **0**.**007**	z = 3.88, ***p*** < **0**.**003**
Oats	χ2 = 63.88, df = 2, ***p*** < **0**.**001**	z = 4.34, ***p*** < **0**.**001**	z = 3.87, ***p*** < **0**.**001**	z = 7.99, ***p*** < **0**.**001**
Turnip rape	χ2 = 76.84, df = 2, ***p*** < **0**.**001**	z = 3.02, ***p*** < **0**.**007**	z = 5.76, ***p*** < **0**.**001**	z = 8.27, ***p*** < **0**.**001**
Potato	χ2 = 5.87, df = 2, *p* < 0.053	z = 0.16, *p* < 0.987	z = 2.16, *p* < 0.078	z = 2.01, *p* < 0.110

Proportion of seeds germinated of total seeds sown (two faba bean seeds and four oat and turnip rape seeds per pot) and the cumulative number of potato sprouts by day and treatments (C = control, G = pure glyphosate, GBH = glyphosate based herbicide). Day (not shown) was highly significant in each model. Statistically significant *p*-values (*p* < 0.05) are in bold.

**Figure 4:**
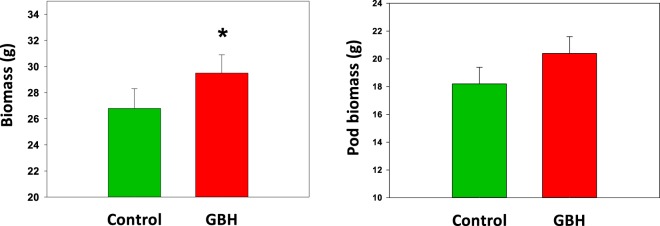
Above-ground biomass and pod biomass (mean ± S.E.) of faba beans growing in soil treated with glyphosate-based herbicide (GBH, Roundup Gold) or in control soil at the end of the field experiment. Figures are drawn using raw data from the experiment. Statistically significant differences between treatments (*p*-values < 0.05) are marked with *.

In the beginning of the field experiment, the faba beans grew equally in GBH and C soils. However, at the end of the experiment, the GBH-treated faba beans were taller (χ2 = 3.99, df = 1, *p* = 0.045) and their biomass greater (χ2 = 3.99, df = 1, *p* = 0.046) compared to plants in C soils (Fig. 4). However, the yield was not affected, as the total weight of bean pods did not differ between C and GBH-treated soils (Fig. 4; χ2 = 2.75, df = 1, *p* = 0.098).

### Oats

In the greenhouse experiment, the control oats germinated clearly faster than oats in G or GBH soil (Fig. 2, Table 1). All differences between the treatments were highly significant. The total germination percentage of oats in GBH soil (47%), G soil (50%) and C soil (62%) differed (χ2 = 6.40, df = 2, p = 0.041), mainly due to borderline differences between the C and GBH soils (z = 2.41, p = 0.058).

The final biomass (Fig. 3) differed between the treatments (biomass: χ2 = 12.72, df = 2, *p* = 0.002), but oats in C or pure G soils grew equally well (Tukey comparison between treatments: C–G: z = 0.26, p = 0.965). Oat biomass was significantly decreased in GBH-treated soil compared to oats in G and C soils (C–GBH: t = 3.24, p = 0.009, G–GBH: t = 3.00, p = 0.016).

**Figure 5:**
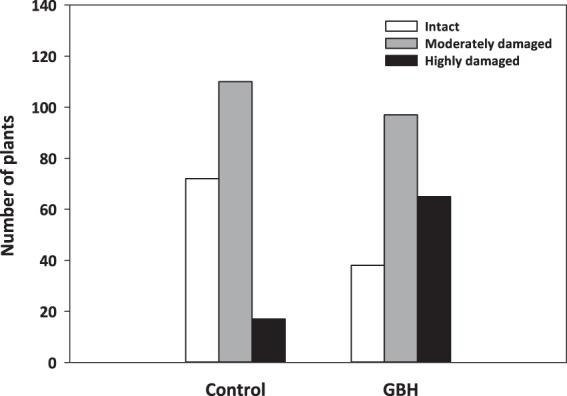
Herbivore damage of oat plants growing in soils treated with glyphosate-based herbicide (GBH, Roundup Gold) or control soil in the field experiment.

In the field experiment, the oats had encountered herbivore grazing by barnacle geese (*Branta leucopsis*) during the first weeks of establishment. The geese were visually observed to visit the experimental area, and their droppings were discovered next to oat plants. The herbivore damage was estimated six weeks after sowing of oat seeds using a three level classification: 0 = not eaten, 1 = moderately damaged, 2 = highly damaged. Herbivore damage was significantly higher in GBH-treated oats compared to C oats (χ2 = 39.2, df = 2, *p* < 0.001) (Fig. 5). Survival of the oat plants was affected by herbivore damage (χ2 = 9.106, df = 2, *p* < 0.011) and GBH treatment (χ2 = 3.89, df = 1, *p* < 0.049), but not their interaction (χ2 = 1.13, df = 2, *p* < 0.570). Survival increased when the plants were not highly damaged (Tukey comparison between damage classes 2 − 1: z = 2.959, *p* < 0.009; classes 1 − 0: z = 0.447, *p* = 0.895).

**Figure 6:**
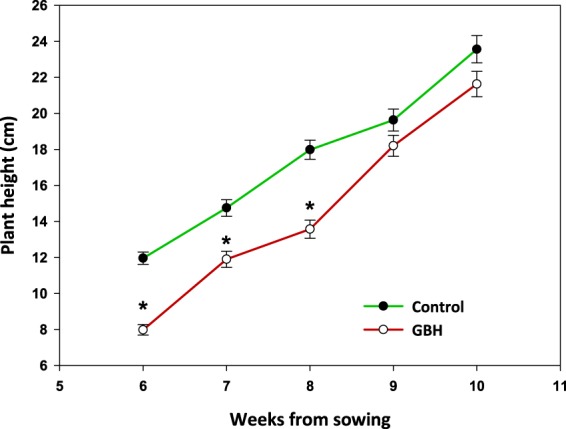
Height (mean ± S.E.) of oats growing in soil treated with glyphosate-based herbicide (GBH, Roundup Gold) or control soil in the field experiment. Figure is drawn using raw data from the experiment. Statistically significant differences between treatments (*p*-values < 0.05) are marked with *.

For the first two weeks after the grazing damage was observed, the non-grazed oat plants (class 0 = not eaten) in C soils were taller than the non-grazed plants in GBH soils (week 6: χ2 = 85.42, *p* < 0.001; week 8: χ2 = 20.518, p < 0.001; df = 1), but thereafter the difference became insignificant (week 9: χ2 = 3.03, *p* = 0.082, df = 1). The same trend in plant height was detected throughout the data including grazed and non-grazed plants (Fig. 6, week 6: treatment χ2 = 40.25, *p* < 0.001, herbivory χ2 = 241.7, *p* < 0.001; interaction χ2 = 0.29, p = 0.591; week 9: treatment χ2 = 0.79, herbivory χ2 = 9.81, *p* < 0.002; interaction χ2 = 0.02, *p* = 0.889).

The number of oat inflorescences in C soil was twice as high as the number of inflorescences in GBH soil (χ2 = 173.48, df = 1, *p* < 0.001). In a back-transformed scale, the oats in C soil were estimated to have 79 inflorescences (95% confidence interval: 52–119 inflorescences) and those in GBH soil to have 37 inflorescences (95% confidence interval: 24–56 inflorescences) per plot.

### Turnip rape

In the greenhouse, turnip rape germinated earlier in C soil than in G soil (z = 7.99, *p* < 0.001), which again was earlier than the germination in GBH soil (z = 3.01, *p* < 0.007) (Table 1, Fig. 2). The total number of germinated seeds at the end of the greenhouse experiment differed between the treatments (χ2 = 14.77, df = 2, *p* < 0.001, Fig. 2). Total germination of C seeds was 47%, G seeds 33% and GBH seeds 17%.

The biomasses of the treatment groups in the greenhouse experiment were different at harvest (Fig. 3; χ2 = 10.63, df = 2, *p* = 0.005), but only the C and GBH treatment differed from each other in pairwise comparisons (G–GBH: t = 1.17, *p* = 0.487; C–G: z = 2.20, *p* = 0.101; C–GBH z = 3.08, *p* = 0.0189, df = 16).

In the field experiment, statistical comparison of turnip rape establishment and growth was not possible due to random and low germination and poor growth of the established plants.

### Potato

In the greenhouse experiment, potatoes in C soil tented to sprout earlier compared to those in GBH soil (Fig. 2) (day included: χ2 = 5.87, df = 2, *p* < 0.053; GBH–C: z = 2.16, *p* = 0.078; G–C: z = 2.01, *p* = 0.110; G–GBH: z = 0.16, *p* = 0.987). Total potato sprouting was not affected by G or GBH treatments (χ2 = 0.45, df = 2, *p* = 0.800)

Potatoes growing in C soil had longer sprouts at the time of the first measurement (5 days after planting: χ2 = 9.65, df = 2, *p* = 0.008; C–GBH: t = 2.97, *p* = 0.016; C–G: t = 2.26, *p* = 0.077; GBH–G: t = 0.71, *p* = 0.761; df = 28). The height of the plants in the C and GBH soils tended to be different on day 20 (χ2 = 5.87, df = 2, *p* = 0.053; C–GBH: t = 2.38, *p* = 0.062; C–G: t = 0.77, *p* = 0.724; GBH–G: t = 1.69, *p* = 0.736; df = 28). However, at the end of the experiment (day 27), shoot biomass was not statistically different between plants grown in C, G and GBH soils (χ2 = 1.27, df = 2, *p* = 0.530, Fig. 3).

**Figure 7:**
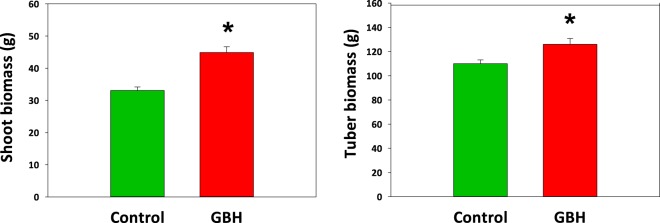
Shoot and tuber biomass (mean ± S.E.) of potatoes growing in soils treated with glyphosate-based herbicide (GBH, Roundup Gold) or control soil at the end of the field experiment. Figures are drawn using raw data from the experiment. Statistically significant differences between treatments (*p*-values < 0.05) are marked with *.

In the field experiment the potato shoot biomass in GBH-treated soils was 25% higher (χ2 = 10.68, df = 2, *p* = 0.001) and the potato tuber yield 14% higher (χ2 = 5.55, df = 2, *p* = 0.018) compared to the potatoes growing in the C soil (Fig. 7).

### Glyphosate residues in soil and plants

Traces of glyphosate or AMPA were not detected in the C soil or control plant samples in the greenhouse or in the field experiments.

Glyphosate residues from prior GBH treatments (years 2014 and 2015) were detected in the soil of GBH plots of the field experiment in May 2016 before the GBH treatments (glyphosate 0.91 mg/kg, AMPA 0.96 mg/kg). At the end of July 2016, the soil contained 0.91 mg/kg of glyphosate and 1.00 mg/kg of AMPA. In the greenhouse experiment, pure G-treated soil contained 1.3 mg/kg of glyphosate and 0.28 mg/kg of AMPA, while the GBH-treated soil contained 3.1 mg/kg of glyphosate and 0.43 mg/kg of AMPA two days after the soil treatments.

Glyphosate and AMPA traces were only detected from potato tubers (glyphosate 0.02 mg/kg and AMPA 0.07 mg/kg) grown in GBH soil, whereas all the other plant samples were free of glyphosate and AMPA.

## Discussion

Our results indicate that the use of GBH as well as surfactants and other ingredients of commercial herbicide products have different effects on the seedling establishment of seed- and vegetative-propagated crops. In all the studied seed-propagated crops, germination was faster, and in turnip rape and oats the total germination percentage was higher in the C soils compared to the pure G- or GBH (Roundup)-treated soils. The aboveground biomass of plants in C soils was also greater than that of those in GBH treated soils at the end of the experiments. In contrast, species with larger endosperms, such as beans and vegetatively propagated potato, appeared to benefit from GBH treatment. The bean germination and sprouting of potato tubers at the very beginning of the experiment tended to be faster in C soils, but at the end of the field experiment the aboveground biomass of the faba beans and the shoot biomass and tuber yield of potatoes was higher in GBH soil than in C soil. The enhanced performance of the potato plants in GBH soils was consistent in both the greenhouse and field experiments.

We assume that these results reflect the differences in the steps of germination, the amount of energy reserves for developing seedlings and the nutrient requirements of the individual species. Germination always requires sufficient moisture conditions. However, we may assume that seeds require more moisture to swell and soften the seed coat than potato nodes (or eyes) on tubers. Thus, seed-propagated crops with limited endosperms as an energy source are likely to be exposed to GBH residues in soils following the imbibition at the beginning of the seed germination. Furthermore, seedlings have limited energy storage in the cotyledon, whereas potato sprouts are more self-sufficient, subsisting primarily on the tuber. This may provide potatoes with a longer GBH-free time window to establish and develop shoots. The accumulation of glyphosate and AMPA traces in potato tubers but not in shoots suggests that potato plants are able to translocate and store the glyphosate and its main metabolites into non-photosynthesizing plant tissues. Avoidance of the negative effects of GBH residues does not, however, explain the better growth and yield of potatoes. One possible explanation for the detected positive effects of GBH-treated soils on potatoes is enhanced phosphorus content in the soils. Glyphosate can play an important role in determining the availability of phosphate to plants in soils because phosphorus and glyphosate share adsorption mechanisms and sites in soils^6,20–22^. If glyphosate outcompetes phosphorus for sorption sites in soils, GBH usage increases the amount of phosphorus available to plants. In particular, among our study plants, potato is known for its relatively high phosphorus requirements^23^.

Our results suggest that the use of GPH may have unintended and undesirable consequences for farmers. The speed of germination and early growth may be crucial for the plants, depending on the abiotic and biotic environmental factors. Especially in spring, earlier individuals may benefit from moisture and a lack of competition^24,25^. Thus, delayed germination and weakened growth of seed-propagated crops in GBH-contaminated soils^26,27^ may invalidate the intended crop protection if targeted weeds get a head start in early spring. This should be taken into account if weeds are not mechanically managed. In addition to stronger competition, delayed and prolonged germination and a prolonged seedling phase may increase the risk of pests and pathogens^28^. For example, damage by flea beetles of the genus *Phyllotreta*, one of the most serious and widespread pests of oilseed and turnip rape, appears to be higher in late-seeded than in earlier-seeded oilseed rape^29,30^. Thus, the use of GBH may increase the yield loss caused by flea beetles and further challenge spring-planted oilseed rape and turnip rape cultivation in Europe, where the use of neonicotinoid pesticides has been banned.

Furthermore, our results suggest that glyphosate can enhance the attractiveness of plants to vertebrate herbivores. In the field experiment, the oat plants growing in GBH-treated experimental plots experienced heavy barnacle geese grazing while the adjacent plants in C plots were only mildly grazed. The difference might be explained by poor growth of GBH oats or changes in their chemotypic traits. Glyphosate is known to inhibit the 5-enolpyruvylshikimate-3-phosphate synthase (EPSPS) in the shikimate acid pathway, thereby interfering with the production of tryptophan, phenylalanine or tyrosine, which are precursors of proteins and other molecules, including growth promoters (e.g., indoleacetic acid, IAA) or secondary compounds with known importance for plant defense against herbivores (e.g., tannins, anthocyanins, flavonoids and lignin)^6,31^. Because we measured the size of the oat plants only after the herbivores had visited the experimental field, we were not able to reveal whether the plants assigned to the GBH and C plots were phenotypically distinguishable from each other such that the geese could visually spot the plants in the GBH plots. However, in the greenhouse and field experiments, the intact oat plants were germinating and growing faster and large in C soils compared to GBH-treated soils, suggesting that the geese were possibly attracted by poorly growing oat plants.

Overall, the effect of pure glyphosate was weaker compared to that of the commercial formulation (Roundup Gold) containing the same amount of glyphosate. This supports other studies^32^ suggesting that other ingredients in GBH, such as surfactants, solvents and preservatives, could also cause adverse effects on non-target organisms.

Our results clearly demonstrate that the use of GBH has detectable effects on crop plant germination and growth, and their quality to herbivores, even though we used field-realistic concentrations of GBH and the experimental plants were introduced into the soil after a two-week withholding period. Referring to seed germination and seeding establishment, GBH negatively and positively affected seedling establishment of seed- and vegetative-propagated crops, respectively. Turnip rape, oat and faba bean germination was significantly delayed in G-treated soils, and in the case of turnip rape and oat, the germination was also decreased. The poor establishment and growth may explain that oats grown in GBH soils attracted barnacle geese to feed on them. In contrast to seed-propagated crops, GBH treatment boosted the growth of vegetatively propagated potatoes, and glyphosate appeared to accumulate in the potato tubers. This leads to the critical question whether the residues in potatoes have consequences for the subsequent year’s yield. These results emphasize the importance of a more comprehensive understanding of the effects of GBH on the productivity of crop plants and their chemical ecology, affecting their pest and pathogen resistance and thus the need for crop protection.

## Data Availability

The datasets generated during and/or analyzed during the current study are available from the corresponding author on reasonable request.
